# Effects of Pain and Depression on ADL Disability Over 6 Years of Follow-Up Among Older Adult Americans

**DOI:** 10.1093/geroni/igaa057.1191

**Published:** 2020-12-16

**Authors:** Jaspreet Sodhi, Soham Al Snih

**Affiliations:** The University of Texas Medical Branch at Galveston, Galveston, Texas, United States

## Abstract

The objective of this study was to examine the effect of co-occurring pain and depression on ADL disability over 6-years of follow-up among older adult Americans. We studied 5,236 participants aged 65 years and older from the National Health and Aging Trends Study (2011-2017) The primary outcome was ADL disability defined as any limitation in ADLs (eating, bathing, transferring, dressing, moving inside, and out of bed). The independent predictors were self-reported pain and depression. Covariates included socio-demographics (age, gender, marital status, race/ethnicity and years of formal education), body mass index, and comorbidities. Participants were categorized into four groups according to pain and depression: no pain and no depression, pain only, depression only, and depression and pain. Generalized Estimation Equation model was used to estimate the odds of ADL disability as a function of pain and depression. All variables were analyzed as time-varying except for age, race/ethnicity, and education. The odds of ADL disability as a function of pain only and depression only was 1.62 (95% CI 1.38-1.91) and 2.13 (95% CI 1.54-2.95), respectively. The odds of ADL disability as a function of pain and depression were 3.92 (95% CI 3.13-4.92). Older age, being married, Hispanics, and comorbid conditions were also predictive factors of ADL disability over time. Female participants and those with higher levels of education were less likely to report ADL disability over time. The findings suggest that both pain and depression significantly increased the risk of ADL disability in this population over 6-years.

